# Design and predictive performance analysis of an early readmission risk index in a mental health hospitalization unit: an exploratory study

**DOI:** 10.3389/fpsyg.2026.1776125

**Published:** 2026-04-23

**Authors:** Yaiza García-Illanes, Vera Carbonell-Aranda, María Traverso-Rodriguez, Gloria Pérez-Guerrero, Antonio Bordallo-Aragón, Jesús Herrera-Imbroda, José Guzmán-Parra

**Affiliations:** 1 IBIMA Plataforma BIONAND, Instituto de Investigación Biomédica de Málaga, Málaga, Spain; 2 Unidad de Gestión Clínica de Salud Mental, Hospital Regional Universitario de Málaga, Málaga, Spain; 3 Facultad de Psicología, Departamento de Personalidad, Evaluación y Tratamiento Psicológico, Universidad de Málaga, Málaga, Spain

**Keywords:** patient readmission, psychiatry, rehospitalization, risk factors, thirty day readmission

## Abstract

Early hospital readmission in mental health inpatients negatively affects patient outcomes and increases healthcare costs, making early risk identification crucial. This study aimed to develop a 30-day readmission risk index using a retrospective case–control design in the Mental Health Hospitalization Unit of the Hospital Regional Universitario de Málaga, including 196 admission episodes (98 early readmissions and 98 controls). Univariate and multivariate logistic regression analyses identified the following predictive variables: previous hospital admissions, presence of a personality disorder, high social risk, having used emergency services in the past year, having a legal capacity restriction, aggressive behavior at admission, and marital status. The resulting index demonstrated good predictive performance (AUC = 0.764), with internal validation showing an optimism-corrected AUC of 0.727 and acceptable calibration (slope = 0.796, intercept = −0.023). This tool may be useful for risk stratification and post-discharge planning, but should be used cautiously due to the exploratory nature of the study.

**Clinical trial registration:**

ClinicalTrials.gov, identifier NCT06604780.

## 1 Introduction

The post-discharge period from a mental health inpatient unit represents a phase of high clinical and social vulnerability, during which patients’ psychological well-being may be compromised. Early hospital readmission is associated with adverse outcomes for both individuals and healthcare systems, including reduced quality of life, clinical deterioration, and loss of social and occupational functioning (Tyler et al., 2019; Müller et al., 2024). Approximately one-third of individuals admitted for mental health reasons are readmitted within a year, with early readmissions—commonly defined as occurring within 30 days of discharge—affecting around 10–15% of patients (Vigod et al., 2015; Ortiz, 2019; Owusu et al., 2022; Carbonell-Aranda et al., 2024). Rates vary across countries, highlighting context-specific factors affecting this phenomenon (Berardelli et al., 2022). Early readmission is widely used as a quality indicator for assessing the continuity of mental healthcare and identifying gaps in post-discharge support (Hariman et al., 2020; Muhammad et al., 2023).

Among the clinical risk factors that may contribute for early readmission, a history of previous hospitalizations stands out as one of the most consistent (Lorine et al., 2015; Donisi et al., 2016; Guzman-Parra et al., 2018; Zhao et al., 2020). Mental health diagnosis has also been associated with the risk of readmission, particularly schizophrenia (Cook et al., 2020; Cabello-Rangel et al., 2024), although other diagnoses such as personality disorder, bipolar disorder, and depressive disorder have also been linked to an increased risk of readmission (Muhammad et al., 2023). Other related factors include the length of hospital stay (Zhang et al., 2011; Vázquez-Real et al., 2022), substance use (Giovanni et al., 2020; Gobbicchi et al., 2021), symptom severity, and the involuntary nature of admission (Müller et al., 2024). Regarding sociodemographic factors, male sex, younger age, marital status, and low socioeconomic level have also been associated with a higher risk of readmission (Fonseca-Barbosa and Gama-Marques, 2023; Müller et al., 2024). However, the findings may vary across settings, populations, and study designs.

Despite the identification of multiple risk factors for early readmission, few studies have developed composite models that allow stratified, personalized interventions. Effective strategies often include post-discharge components, such as follow-up visits or phone calls, to ensure continuity of care, treatment adherence, and intensive case management (Owusu et al., 2022; Tyler et al., 2023). Existing tools, mainly developed in English-speaking contexts, show moderate predictive capacity and are limited in their generalizability to diverse clinical and sociocultural settings (Gearing et al., 2009; Vigod et al., 2015; Tulloch et al., 2016; Hariman et al., 2020). Consequently, there is a clear need for practical and validated methods for early risk stratification specifically adapted to Spanish mental health services.

In response to this need, an Early Readmission Risk Index has been developed as part of the Readmission Prevention Program (Carbonell-Aranda et al., 2025) implemented in the Mental Health Hospitalization Unit, in order to identify inpatients with higher clinical and social vulnerability and to guide targeted post-discharge interventions, with the aim of improving patient outcomes and reducing early hospital readmissions.

## 2 Methodology

### 2.1 Study design

A retrospective, observational case–control study was conducted through the consultation of admission records and review of medical charts to obtain information from the year prior to admission. This analysis corresponds to the index-development phase of a broader transition-to-discharge program registered at ClinicalTrials.gov (NCT06604780).

### 2.2 Procedure

An interdisciplinary working group was formed, including professionals with expertise in mental health, both in clinical practice and in the design of continuity-of-care strategies. The team convened to discuss early psychiatric readmissions and identify clinically and socially relevant variables. A literature review was conducted to compile a list of potentially important variables. Subsequently, patient records from the acute psychiatric unit were reviewed, and variables of interest were selected for extraction. Data from the Andalusian Health Service electronic medical records system (DIRAYA) were also included. Based on these steps, a final set of variables was selected to design the risk index. The final tool is provided in full in the Supplementary material.

### 2.3 Study setting

The study was conducted in the Mental Health Hospitalization Unit (MHHU) of the Hospital Regional Universitario de Málaga (HRUM), a mental health service within a public general hospital managed by the Andalusian Health Service. The MHHU is part of the HRUM Mental Health Clinical Management Unit and serves as a reference for the regional hospitals of Málaga Axarquía Este and Málaga Norte Antequera. According to 2022 data from the Andalusian Mental Health Program, the HRUM Clinical Management Unit serves 336,968 people, while the regional units cover 150,823 and 109,958 individuals, totaling a reference population of 597,749. The MHHU has 42 beds in double rooms, with admissions coming from the emergency department following psychiatric evaluation or scheduled admissions from other units. The average occupancy in 2022 was 28 beds, approximately two-thirds of capacity, with an average length of stay of 13.7 days. In the same year, the 30-day readmission rate recorded at MHHU was 15.08%.

### 2.4 Participants

All admissions from January 1 to December 31, 2021, were included. Episodes corresponded to adult patients within the HRUM catchment area. The sample for the study consisted of readmissions occurring within the first 30 days after discharge (n = 98). To ensure comparability between groups and stability in the regression models, a control group (n = 98) was randomly selected from patients who did not experience readmission during the same period.

### 2.5 Ethical considerations

The study protocol was approved by the Provincial Research Ethics Committee of Málaga (SICEIA: PPREINGRESOTEMPRANO). The committee authorized the study to be conducted without the informed consent of participants, as it was a retrospective observational study based on clinical data generated through routine care, with confidentiality maintained at all times. The study adhered to the ethical principles established in the Declaration of Helsinki of the World Medical Association (latest revision, 2013), the European Parliament and Council Regulation (EU) 2016/679 of April 27, 2016, on the protection of natural persons regarding the processing of personal data and the free movement of such data (General Data Protection Regulation), and the Spanish Organic Law 3/2018 of December 5 on the Protection of Personal Data and guarantee of digital rights.

### 2.6 Description of variables

#### 2.6.1 Dependent variable (VD)

The dependent variable was early readmission, defined as the occurrence of a new hospital admission within 30 days after discharge.

#### 2.6.2 Independent variables (IVs)

##### 2.6.2.1 Sociodemographic variables

Included age, marital status (single/separated or married/widowed), and employment status (employed or unemployed). The gender was excluded from the analytical models, as intentional matching was applied during sample selection to ensure equitable gender distribution between groups. This methodological decision minimized analytical bias due to unequal gender representation and avoided interpretations that could lead to gender-biased preventive interventions. Controls were matched only by sex. All other variables were considered for inclusion in the risk index and were retained or excluded according to the predefined selection criteria.

##### 2.6.2.2 Social variables

The following variables were included:

- Social risk: was operationalized as a pragmatic screening measure for routine clinical use. A committee of experts standardized five key domains based on a targeted literature review and clinical relevance: (1) family situation (living alone or with a dependent family member); (2) economic situation (earning below minimum wages or receiving only non-contributory pensions); (3) housing (inadequate housing conditions); (4) social relationships (minimal or no social contacts); and (5) social network (lack of external support when needed). Social risk was considered present if at least one domain was affected, allowing the study to capture meaningful social vulnerabilities consistently.
- Legal capacity restriction: indicated whether a current legal ruling limited full or partial decision-making capacity due to a legal incapacitation process. Before the enactment of *Law 8/2021*, the Spanish legal framework allowed judicial incapacitation and guardianship, whereby a judge could partially or totally restrict an individual’s legal capacity and appoint a guardian. These provisions were in force during the study’s data collection period.
- Criminal history: recorded if the patient’s medical record documented prior offenses, legal proceedings, arrests, or other criminal behavior.

##### 2.6.2.3 Clinical variables

###### 2.6.2.3.1 Personal history

Personal history variables included “mental health history,” coded as present when the medical record documented that the patient had previously received care or treatment in any mental health service or unit, regardless of the type of professional involved. In addition, previous admissions to the MHHU, emergency care visits within the 12 months prior to admission, and history of suicide attempts were documented. The dichotomous variable “Somatic comorbidity” was coded as present if the patient had at least one chronic medical condition requiring long-term follow-up and ongoing symptom management (e.g., diabetes mellitus, hypertension, chronic obstructive pulmonary disease, or cardiovascular disease). Acute conditions (e.g., infections or trauma) were excluded. Information was obtained from admission and discharge reports and, when necessary, verified through review of the complete medical record.

###### 2.6.2.3.2 Adherence-related variables

- Adherence to CMHU follow-up: adherence was calculated over the 12 months prior to admission as the proportion of scheduled appointments attended (attended appointments ÷ total scheduled × 100). A score of 100% indicated full adherence and 0% indicated total non-attendance. For clinical interpretability, adherence was dichotomized: patients were classified as adherent if they attended ≥80% of appointments, and non-adherent if they attended <80%.
- Medication adherence: identified as treatment discontinuation or incorrect use during the month prior to admission, based on admission or discharge reports and CMHU records.
- Nursing follow-up attendance: considered adequate if the patient attended at least two nursing consultations at the CMHU within the six months preceding admission.

###### 2.6.2.3.3 Clinical diagnoses

Primary diagnoses were categorized according to ICD-10 as: F10–F19 (mental and behavioral disorders due to psychoactive substance use), F20–F29 (schizophrenia, schizotypal and delusional disorders), F30–F39 (mood disorders), F40–F49 (neurotic, stress-related and somatoform disorders), F60–F69 (personality and adult behavioral disorders). Secondary diagnoses included the above categories plus “other ICD-10 diagnoses” and “no secondary diagnosis.” Substance use was recorded both when formally diagnosed (F10–F19) and whenever documented in the clinical record, irrespective of diagnostic coding.

###### 2.6.2.3.4 Admission-related variables

Information was obtained from various sources, including the admission report, immediately preceding emergency episode, or the mental health team’s clinical record, as available. Included admission route (emergency or scheduled), admission type (voluntary or involuntary), and length of stay (in days). Two variables related to violent behaviors prior to admission were collected: self-directed and other-directed. In addition, the presence of deficits in self-care was recorded, affecting daily activities such as personal hygiene, feeding, sleep, mobility, and social relationships. Variables related to pre-discharge planning were recorded, including prescription of depot injectable medication only if the patient was discharged with this treatment. Coordination with the CMHU was also documented, if recorded in the hospitalization unit discharge summary or the CMHU clinical record.

### 2.7 Data analysis

Descriptive analyses were conducted for continuous variables (mean ± SD or median and interquartile range if not normally distributed) and categorical variables (frequencies and percentages). Univariate logistic regression was performed to explore associations between independent variables and early readmission, and variables meeting the initial inclusion criteria were considered for the multivariate model using a stepwise forward selection approach. Since the aim of the study was to develop a predictive tool for psychiatric readmission, a relatively inclusive covariate selection strategy was employed. In the multivariable phase, a retention threshold of *p* < 0.20 was used to avoid the premature exclusion of variables that may contribute meaningful predictive information in a multifactorial clinical phenomenon. This approach prioritized the identification of potential predictive signals and the capture of heterogeneity within the clinical population, rather than adherence to a strictly confirmatory criterion. The decision reflected the exploratory and predictive nature of the study, where the goal was to develop a broadly applicable risk index rather than to establish causal relationships. It was also considered clinically reasonable in light of the intended use of the model, namely to support the identification of patients who may benefit from additional follow-up. To identify the most robust predictors, a second model was fitted using a more restrictive inclusion criterion of *p* < 0.10.

Model discrimination was evaluated using the area under the receiver operating characteristic curve (AUC), and sensitivity and specificity were calculated. Internal validation of the predictive model was performed using bootstrap resampling (1,000 iterations) to estimate optimism in model performance. Both the apparent and optimism-corrected AUC values were calculated. Model calibration was also assessed by estimating the calibration slope and calibration intercept. All analyses were performed using R (version 4.4.2).

The index was developed following Sullivan’s approach, which converts the logistic regression model into a clinically applicable points-based tool while preserving its predictive accuracy (Sullivan et al., 2004). In this procedure, regression coefficients from the multivariate logistic model were estimated, and each risk factor was categorized with a reference value assigned. The distance of each category from its reference was then used to assign points to the corresponding variable, and these points were subsequently summed to calculate the total score for each patient. Further details of the point-score derivation are described in Sullivan et al. (2004).

## 3 Results

In the univariate analysis, several sociodemographic and clinical characteristics were significantly associated with early readmission: primary diagnosis (Personality Disorder vs. Substance Use Disorder; OR = 4.55, *p* = 0.007), presence of a personality disorder (OR = 2.88, *p* = 0.017), previous admissions (OR = 4.44, *p* < 0.001), mental health history (OR = 2.81, *p* = 0.001), emergency visits in the previous year (OR = 2.11, *p* = 0.024), social risk (OR = 2.11, p = 0.024), and adherence to follow-up at the CMHU during the previous year (OR = 1.95, *p* = 0.026). Details of the univariate analysis are shown in Table 1.

**Table 1:** Univariate analysis of factors associated with early readmission.

Variables	Total admissions	Early readmission	No early readmission	OR	95% CI	*p*-value
	(*N* = 196)	(*N* = 98)	(*N* = 98)			
Sociodemographic variables						
Age, mean (SD)	42.84 ± 14.14	42.02 ± 13.59	43.66 ± 14.70	0.99	0.97–1.01	0.416
Marital status, *n* (%)						
Single/separated (ref)	141 (83.90)	66 (84.60)	75 (83.30)			
Married/widowed	27 (16.10)	12 (15.40)	15 (16.70)	0.90	0.39–2.08	0.822
Employment, *n* (%)						
No (ref)	153 (78.06)	80 (81.63)	73 (74.49)			
Yes	43 (21.94)	18 (18.37)	25 (25.51)	0.66	0.33–1.30	0.229
Social variables						
Legal capacity restriction, *n* (%)						
No (ref)	171 (87.24)	81 (82.65)	90 (91.84)			
Yes	25 (12.76)	17 (17.35)	8 (8.16)	2.36	0.99–6.06	0.059
Social risk, *n* (%)						
No (ref)	52 (26.53)	19 (36.50)	33 (63.50)			
Yes	144 (73.47)	79 (54.90)	65 (45.10)	2.11	1.11–4.11	0.024
Criminal history, *n* (%)						
No (ref)	158 (80.61)	77 (78.57)	81 (82.65)			
Yes	38 (19.39)	21 (21.43)	17 (17.35)	1.30	0.64–2.67	0.471
Variables related to personal history						
Mental health history, *n* (%)						
No (ref)	135 (68.88)	78 (79.59)	57 (58.16)			
Yes	61 (31.12)	20 (20.41)	41 (41.84)	2.81	1.50–5.38	0.001
Previous admissions, *n* (%)						
No (ref)	67 (34.18)	18 (26.87)	49 (73.13)			
Yes	129 (65.82)	80 (62.02)	49 (37.98)	4.44	2.36–8.66	<0.001
History of suicide attempts, *n* (%)						
No (ref)	115 (58.67)	53 (54.08)	62 (63.27)			
Yes	81 (41.33)	45 (45.92)	36 (36.73)	1.46	0.83–2.60	0.192
Emergency visits in the previous year, *n* (%)						
No (ref)	52 (26.53)	19 (36.50)	33 (63.50)			
Yes	144 (73.47)	79 (54.90)	65 (45.10)	2.11	1.11–4.11	0.024
Somatic comorbidity, *n* (%)						
No (ref)	105 (53.57)	54 (55.10)	51 (52.04)			
Yes	91 (46.43)	44 (44.90)	47 (47.96)	0.88	0.50–1.55	0.667
Nursing follow-up in the 6 months prior, *n* (%)						
No (ref)	140 (71.42)	72 (73.47)	68 (69.39)			
Yes	56 (28.57)	26 (26.53)	30 (30.61)	0.82	0.44–1.52	0.527
Medication adherence in the month prior, *n* (%)						
Non-adherent (ref)	81 (41.30)	43 (43.90)	55 (56.10)			
Adherent	115 (58.70)	38 (38.80)	60 (61.20)	1.23	0.70–2.19	0.469
Follow-up adherence at the CMHU in the previous year, *n* (%)						
Non-adherent (ref)	71 (36.20)	43 (43.90)	28 (28.60)			
Adherent	125 (63.80)	55 (56.10)	70 (71.40)	1.95	1.09–3.56	0.026
Variables related to mental health diagnosis						
Primary diagnosis, *n* (%)						
Substance use (F10-19) (ref)	31 (17.32)	11 (12.50)	20 (21.98)			
Schizophrenia and related disorders (F20-29)	67 (37.43)	31 (35.23)	36 (39.56)	1.57	0.66–3.86	0.317
Mood disorders (F30-39)	42 (23.46)	21 (23.86)	21 (23.07)	1.82	0.71–4.82	0.218
Phobic anxiety disorders (F40-49)	11 (6.15)	5 (5.68)	6 (6.59)	1.52	0.36–6.21	0.559
Personality disorders (F60-69)	28 (15.64)	20 (22.73)	8 (8.79)	4.55	1.56–14.33	0.007
Secondary diagnosis, *n* (%)						
Substance use (F10-19) (ref)	22 (11.22)	10 (45.45)	12 (54.55)			
Schizophrenia and related disorders (F20-29)	5 (2.55)	4 (80.00)	1 (20.00)	4.80	0.59–102.31	0.19
Mood disorders (F30-39)	13 (6.63)	7 (53.85)	6 (46.15)	1.40	0.35–5.71	0.632
Phobic anxiety disorders (F40-49)	13 (6.63)	6 (46.15)	7 (53.85)	1.03	0.25–4.12	0.968
Personality disorders (F60-69)	13 (6.63)	5 (38.46)	8 (61.54)	0.75	0.18–3.01	0.687
No diagnosis	24 (12.24)	11 (45.83)	13 (54.17)	1.02	0.32–3.28	0.979
Other diagnoses (out of range F10–F69)	106 (54.08)	55 (51.89)	51 (48.11)	1.29	0.52–3.31	0.583
Personality disorder diagnosis, *n* (%)						
No (ref)	168 (85.71)	78 (79.59)	90 (91.84)			
Yes	28 (14.29)	20 (20.41)	8 (8.16)	2.88	1.24–7.30	0.018
History of substance use, *n* (%)						
No (ref)	87 (44.39)	42 (42.86)	45 (45.92)			
Yes	109 (55.61)	56 (57.14)	53 (54.08)	1.13	0.64–1.99	0.666
Variables related to admission						
Self-directed violence, *n* (%)						
No (ref)	132 (67.35)	63 (64.29)	69 (70.41)			
Yes	64 (32.65)	35 (35.71)	29 (29.59)	1.32	0.73–2.42	0.361
Heteroaggressive behaviours at admission, *n* (%)						
No (ref)	120 (61.22)	54 (55.10)	66 (67.35)			
Yes	76 (38.78)	44 (44.90)	32 (32.65)	1.68	0.94–3.02	0.079
Self-care deficit, *n* (%)						
No (ref)	91 (46.42)	45 (45.92)	46 (46.94)			
Yes	105 (53.57)	53 (54.08)	52 (53.06)	1.04	0.59–1.83	0.886
Admission type, *n* (%)						
Involuntary (ref)	130 (66.33)	66 (67.35)	64 (65.31)			
Voluntary	66 (33.67)	32 (32.65)	34 (34.69)	0.91	0.50–1.65	0.762
Admission route, *n* (%)						
Scheduled (ref)	40 (20.41)	16 (16.33)	24 (24.49)			
Emergency	156 (79.59)	82 (83.67)	74 (75.51)	1.66	0.83–3.42	0.159
Length of stay (days, median, IQR)	9 (5–15)	8.5 (5–15)	9 (5–15)	1	0.98–1.04	0.597
Depot injectable medication at discharge (%)						
No (ref)	142 (72.45)	69 (70.41)	73 (74.49)			
Yes	54 (27.55)	29 (29.59)	25 (25.51)	1.23	0.66–2.31	0.523
Coordination with CMHU prior to discharge, *n* (%)						
No (ref)	69 (35.2)	32 (32.65)	37 (37.76)			
Yes	127 (64.8)	66 (67.35)	61 (62.24)	1.25	0.70–2.26	0.455

In the multivariate model, the following variables remained: previous admissions (OR = 4.08, *p* < 0.001), personality disorder diagnosis (OR = 3.80, *p* = 0.010), emergency care in the last year (OR = 1.82, *p* = 0.140), social risk (OR = 2.77, *p* = 0.030), legal capacity restriction (OR = 2.15, *p* = 0.144), heteroaggressive behaviors as reason for admission (OR = 1.61, *p* = 0.144), and marital status (OR = 3.00, *p* = 0.056). Details of the multivariate model are presented in Table 2.

**Table 2:** Predictor variables included in the multivariate model of the index.

Variables	Estimate	Std. error	*z*-value	OR	95% CI	*p*-value
Intercept	−2.972	0.65	−4.58	-	-	<0.001
Previous admissions	1.407	0.39	3.56	4.08	1.92–9.13	<0.001
Personality disorder diagnosis	1.336	0.53	2.50	3.80	1.39–11.49	0.010
Emergency care (last 365 days)	0.601	0.41	1.47	1.82	0.83–4.13	0.140
Social risk	1.020	0.47	2.17	2.77	1.13–7.29	0.030
Legal capacity restriction	0.766	0.52	1.46	2.15	0.78–6.24	0.144
Heteroaggressive behaviors (at admission)	0.479	0.37	1.30	1.61	0.79–3.35	0.193
Marital status Single/separated (ref) Married/widowed	1.097	0.58	1.90	3.00	1.00–9.70	0.056

Following Sullivan’s procedure, a point value was assigned to each variable. The point value corresponded to the truncated OR or an approximate value for ordinal variables. Points assigned to each variable are shown in Table 3.

**Table 3:** Points assigned to variables included in the predictive model.

Variables	Assigned score
Previous admissions (OR = 4.08)	4
Personality disorder diagnosis (OR = 3.80)	4
Emergency care in the previous year (OR = 1.82)	2
Social risk (OR = 2.77)	3
Legal capacity restriction (OR = 2.15)	2
Heteroaggressive behaviors at admission (OR = 1.61)	2
Married or widowed marital status (OR = 3.00)	3

**Figure 1:**
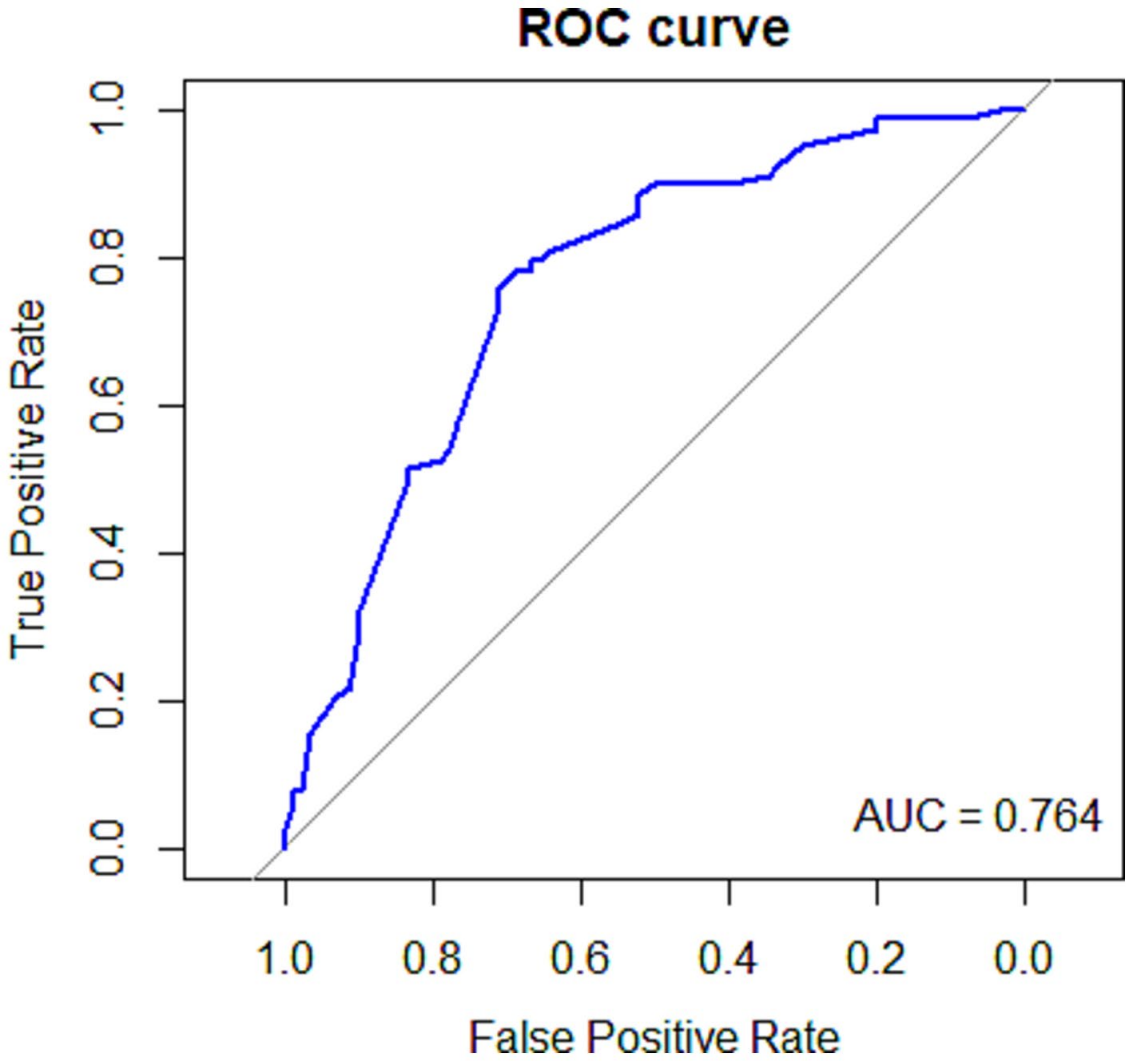
ROC curve of the predictive model.

To further evaluate model performance, ROC curve analysis was conducted. The area under the curve (AUC) was 0.764, indicating good discriminative performance (see Figure 1). Internal validation showed an optimism-corrected AUC of 0.727, supporting the model’s discriminative performance. Model calibration was also acceptable, with a calibration slope of 0.796 and a calibration intercept of −0.023, demonstrating adequate agreement between predicted and observed probabilities.

A stricter multivariate model was also evaluated and the following variables remained in the model: previous admissions (OR = 4.17, *p* < 0.01), presence of a personality disorder (OR = 3.50, *p* = 0.015), social risk (OR = 3.00, *p* = 0.016), and marital status (married/widowed) (OR = 2.52, *p* = 0.096). Details are provided in Supplementary Table 1. Finally, the ROC curve was generated for the stricter model, yielding an AUC of 0.709 and indicating acceptable predictive performance (see Supplementary Figure 1 for details).

### 3.1 Readmission risk calculation

Once the index was obtained, total scores were categorized using percentiles, establishing a three-level classification of readmission risk: low-risk group (score 0–6), intermediate-risk group (score 7–10), and high-risk group (score ≥11).

## 4 Discussion

### 4.1 General interpretation and predictive capacity

The index was developed including seven variables identified in the multivariate model, selected to capture potential factors associated with early readmission. The full model showed a moderate ability to discriminate between patients with and without early readmission. The full model showed moderate discrimination and acceptable calibration. Despite the limitations of the study, the final risk index could have potential clinical utility in supporting the stratification of patients according to their risk of early readmission and informing post-discharge decision-making. Low-risk patients could benefit from standard follow-up, intermediate-risk patients reinforced contact and early outpatient review, and high-risk patients intensive case management, including follow-up calls and coordinated transitions to community mental health services and consideration of participation in a post-discharge group psychotherapy support intervention (Carbonell-Aranda et al., 2025). This stepped approach aims to optimize the allocation of hospital and community resources according to individual risk profiles.

### 4.2 Sociodemographic variables

Marital status showed a marginal association with early readmission in our study, with married or widowed patients presenting a higher estimated risk. Previous studies have generally reported the opposite pattern, with unmarried individuals showing lower risk compared to those who are married or living with a partner (Donisi et al., 2016; Muhammad et al., 2023). These differences may reflect contextual or cultural factors, such as social or family dynamics, and highlight that associations observed in our population may not fully align with findings from other settings. Given the relatively small sample size and the single-center retrospective design, this finding should be interpreted with caution. External validation in larger and more diverse cohorts would be necessary before drawing broader conclusions.

### 4.3 Social variables

In our study, overall social vulnerability was associated with a higher risk of early psychiatric readmission. Previous studies have highlighted the importance of social isolation in increasing readmission risk, while social integration is considered a protective factor (Zhao et al., 2020; Fonseca-Barbosa and Gama-Marques, 2023). Social conditions, such as limited support as observed in populations experiencing homelessness, have been associated with a higher risk of readmission and poorer functional outcomes (Herrera-Imbroda et al., 2023). Individuals with social difficulties are also more likely to be subjected to interventions involving coercive measures. In this context, it has been shown that mechanical restraint combined with involuntary medication is associated with lower functional improvement and an increased likelihood of psychiatric readmission (Herrera-Imbroda et al., 2025).

### 4.4 Clinical variables

Previous admissions emerged as the strongest predictor of early readmission (Lorine et al., 2015; Donisi et al., 2016; Guzman-Parra et al., 2018; Zhao et al., 2020). Personality disorder was also associated with increased risk (Besch et al., 2023; Carbonel-Aranda et al., 2024). Emergency visits in the year prior to admission were relevant predictors, highlighting the role of psychiatric crises, while heteroaggressive behaviors during admission also contributed to higher readmission risk (Di Lorenzo et al., 2016; Tulloch et al., 2016; Lebrat et al., 2024).

Other factors, including substance use, prior suicide attempts, length of stay, and involuntary admission, did not show significant associations in the multivariate analysis, despite being reported as risk factors in other studies (Dias Neto and da Silva, 2008; Zhang et al., 2011; Giovanni et al., 2020; Müller et al., 2024). Physical comorbidity has also been associated with early readmission in previous studies (Šprah et al., 2017; Yang et al., 2020; Selmer et al., 2025). In our study, the fact that different medical conditions were not distinguished may have limited the ability to assess the impact of individual conditions on early readmission risk.

### 4.5 Limitations and future perspectives

These findings should be interpreted cautiously due to limitations inherent to the retrospective, single-center observational design, including potential information loss and the inability to establish causal relationships. The sample size and representativeness limit generalizability, emphasizing the need for replication in larger, diverse populations. Certain observations, such as the association between marital status and readmission risk, may be context-specific, and caution is also warranted when extrapolating to settings with different healthcare structures or resource constraints, where unmeasured contextual factors—such as patients’ socioeconomic background or education—may influence outcomes. In addition, social risk was assessed using an expert-consensus approach rather than a previously validated instrument, which may have introduced some misclassification and limited generalizability. The binary definition (≥1 affected domain) may also reduce sensitivity to severity. The relatively inclusive variable selection strategy may also have increased the risk of over-inclusion, although this was considered acceptable given the predictive aim of the study and the low-risk nature of the intended intervention. Future research may incorporate more detailed social and clinical measures, including patient-reported outcomes, which may help refine predictive models (Yamaguchi et al., 2024).

Furthermore, the exploratory nature of this study should be acknowledged. Some variables included in the final risk index did not reach conventional levels of statistical significance in the multivariate model but were retained based on a liberal selection threshold. This inclusive approach may introduce some degree of statistical uncertainty, and the index should therefore be interpreted as preliminary. External validation in larger samples is required to confirm and refine the model.

## 5 Conclusion

The proposed index integrates seven clinically and socially relevant variables to predict early psychiatric readmissions, reflecting the multifactorial complexity of readmission risk. Considering the variables included in the final model allows a more comprehensive stratification of patients at discharge, consistent with the exploratory aims of the study and the inclusion of multiple predictors to reflect clinical heterogeneity, while accepting some reduction in predictive accuracy. This index could support tailored preventive interventions, improving patient outcomes and optimizing resource allocation. Findings should be interpreted with caution due to the retrospective design and sample size, and future studies should validate the model in larger, diverse populations while exploring additional complementary factors.

## Data Availability

The raw data supporting the conclusions of this article will be made available by the authors, without undue reservation.
