# Supplementary material for: Design and predictive performance analysis of an early readmission risk index in a mental health hospitalization unit: an exploratory study
Source: Front Psychol. 2026 Apr 23;17:1776125. doi: 10.3389/fpsyg.2026.1776125 (PMC13149100; doi:10.3389/fpsyg.2026.1776125)
Supplement: Supplementary file 1 [file Data_Sheet_1.PDF]

Supplementary material

Supplementary Table 1. Strict model with the most predictive variables

	Estimate	Std. Error	z-value	OR	95% CI	p-value
Intercept	-2.274	0.53	-4.27	-	-	<0.001
Previous admissions	1.428	0.39	3.67	4.17	1.99-9.21	<0.001
Personality disorder diagnosis	1.254	0.51	2.42	3.50	1.32-10.31	0.015
Social risk	1.099	0.45	2.40	3.00	1.26-7.68	0.016
Married or widowed marital status	0.925	0.56	1.66	2.52	0.87-7.86	0.096

Supplementary Table 2. Early Readmission Risk Index

**Items**	Check the boxes if the answer is "yes"
1. Has the person had any known previous admissions to a mental health inpatient unit?	Yes (4 points)<br>No
2. Is the person's primary diagnosis upon admission a Personality Disorder (of any type)?	Yes (4 points)<br>No
3. Has the person attended any Emergency Department within the 365 days prior to admission for a reason different from the current admission?	Yes (2 points)<br>No
4. Does the person present social impairment, understood as a deficit in at least one of the following areas of social functioning: family and economic situation, housing, social relationships, or social support network?	Yes (3 points)<br>No
5. Does the person have any legally recognized restriction of legal capacity?	Yes (2 points)<br>No
6. Is the reason for admission related to having exercised violence towards others?	Yes (2 points)<br>No
7. Is the person's marital status married or widowed?	Yes (3 points)<br>No
**Total score:**<br>**Score 0–6:** Low risk<br>**Score 7–10:** Intermediate risk<br>**Score ≥11:** High risk	

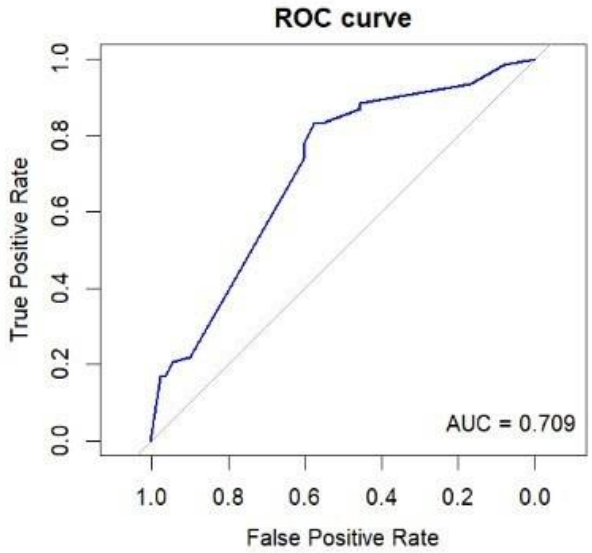

Supplementary Figure 1. ROC Curve of the strict model
